# Determinants of early initiation of breastfeeding in rural Niger: cross-sectional study of community based child healthcare promotion

**DOI:** 10.1186/s13006-017-0134-9

**Published:** 2017-09-29

**Authors:** Naoko Horii, James Allman, Yves Martin-Prével, Dominique Waltisperger

**Affiliations:** 1 Independent Consultant in Behavior Change Communication, Maternal Child Health and Nutrition, 39 rue Buffon, 75005 Paris, France; 2 Population and Development Center (CEPED), Université Paris Descartes, 45 rue des Saints-Pères, 75006 Paris, France; 3 Nutripass Research Unit, Institute of Research for Development (IRD), 44 boulevard de Dunkerque, 13572 Marseille, France; 4 National Institute of Demographic Studies (INED), 133 Boulevard Davout, 75020 Paris, France

**Keywords:** Determinants, Breastfeeding, Behavior change, Community health

## Abstract

**Background:**

Most child deaths are preventable and caused by behaviorally modifiable factors. By promoting optimal breastfeeding, we can reduce neonatal and child mortality risks by 45%. This paper provides new family and community based perspectives to identify factors interfering with the program impact on promoting early initiation of breastfeeding among the most vulnerable populations in rural Niger.

**Methods:**

A secondary analysis of a retrospective cross-sectional study evaluated a UNICEF behavior change program on child healthcare. The study sample is based on a post-hoc constitution of two groups exposed and unexposed to the program. All women (*n* = 1026) aged 14–49 years having at least one child below 24 months of age were included. We measured crude and adjusted odds ratios with chi-square and multivariate logistic regression models.

**Results:**

Independent variables shown to be associated with early breastfeeding include sales activities compared to household work with no direct income (AOR 7.7; 95% CI 1.3, 47.8) and mutual decision for harvest use (AOR 8.6; 95% CI 2.0, 36.8). Antenatal care did not modify the timing of breastfeeding initiation.

**Conclusions:**

A high risk group of mothers with social and economic vulnerability are prone to suboptimal breastfeeding within the first hour of birth. Support from family and neighbors positively influenced early breastfeeding. Those who had no direct income and limited access to health services were a high-risk group, prone to delayed initiation of breastfeeding.

## Background

There is much higher mortality in Sub-Saharan Africa where one child in twelve dies before the 5th year compared to the developed world of one in one hundred and forty seven (UNICEF, 2015). We know that breastfeeding dramatically reduces neonatal mortality risks if newborns are put to breast immediately after birth [1–3]. Recently, an individual behavior, breastfeeding among others, has been globally recognized as a key indicator of health outcome of populations. Social determinants became important to analyze health behavior since socio-economic, cultural and environmental factors could have significant effects on an individual behavior change. Knowing the social determinants of health may address the gaps in individual behavior and accessibility to healthcare [4]. Yet few studies have identified the protective and risk factors affecting breastfeeding in Sub-Saharan Africa.

Income poverty impairs newborn care including the early initiation of breastfeeding [5, 6]. It inhibits knowledge of the benefits of breast milk and delays the initiation of breastfeeding after birth. The socio-economic status of mothers can be defined by two types of resources: individual assets such as income, occupation, education, and community assets such as housing, access to and availability of food and availability of transportation [4]. The relationship between early breastfeeding and educational attainment remains inconclusive. Some studies showed higher education levels of mothers positively associated with early initiation of breastfeeding [6–10] whereas other studies show no significant association [11]. Although parity does not alter early breastfeeding [12, 13], other studies showed that a primiparas, with a lack of previous breastfeeding experience and being of a young age, were major risk factors for delayed initiation of breastfeeding [14, 15]. Household structures in Sub-Saharan Africa characterized by the large extension of inter-generation and co-resident women of childbearing age, all impact neonatal care [16].

The original article presented typology analysis of a UNICEF behavior change communication (BCC) program promoting child healthcare [17]. It showed that some types of the BCC activities had limited impact on promoting the early initiation of breastfeeding among socio-economically deprived mothers in rural Niger. The present research article seeks to identify who are at high risk of delayed initiation of breastfeeding. Understanding the risk factors that lead to suboptimal breastfeeding after birth, is a first step to highlight problems that the program failed to address in its interventions.

## Methods

### Study design

This is a secondary analysis of a cross-sectional retrospective study conducted in 2011 to measure the effect of a UNICEF-led pilot program of behavior change communication (BCC) program promoting child health care [17]. The program began initially in 2008 in two regions of Niger, Maradi and Zinder. The original datasets are derived from three surveys: individual women’s survey, household survey and community survey. These were merged for this secondary analysis.

### Sampling method and study population

This evaluation study is based on a non-randomized purposive sample of 50 villages selected for piloting BCC program activities. In addition to these villages, we added another 25 villages that were not covered by the program at the time of data collection to constitute two groups: exposed and unexposed in a retrospective manner. Within each village purposively selected, 15 households and women were selected based on a two-stage stratified random sampling with no probability proportional to size. The study population for the secondary analysis was extracted from the original dataset of the survey and included all female household members aged 15–49 years who have at least one child below 24 months, currently pregnant or not. The total number of the study population of this secondary analysis is 1026.

### Measurement of outcome variables

Timing of giving the first breast milk after birth is a major outcome variable. The definition of the initiation of breastfeeding as early or delayed is not consistent [18], and the cut-off points to define “early” initiation of breastfeeding vary in previous studies from immediately to three days after birth. We opted for the threshold of the first hour of birth and any practice beyond this is defined as “delayed initiation of breastfeeding” for the following reasons. First, recent WHO/UNICEF guidelines for breastfeeding harmonize their message to promote the start of breastfeeding within the first hour of birth [19–21]. Second, the BCC program promoted initiating breastfeeding within the first hour of birth among other child health care practices. Using the consistent threshold allows us to interpret the findings and apply recommendations within the program context.

### Independent variables

In light of the protective and risk factors shown to determine the timing of initiating breastfeeding after birth in previous studies in Sub-Saharan Africa, we examined a number of socio-economic, demographic, behavioral and environmental factors thought to be related with early initiation of breastfeeding. These independent variables describe characteristics of the interviewed mothers, their interactions with family and community members and access to healthcare services.

### Data analysis

Chi-square tests, bivariate and multivariate logistic regression were used to calculate unadjusted and adjusted odds ratio with STATA SE/13.1 using 95% confidence interval for each independent variable. First, a descriptive analysis depicts each selected variable per program exposure. Second, we measured odds of early initiation of breastfeeding based on the entire study sample adjusted for the program exposure and confounding variables associated with early breastfeeding.

## Results

Apart from parity, the study population showed a significant contrast in its preselected socio-economic and demographic characteristics when comparing the exposed and unexposed groups (Table 1). The proportion of mothers with no education in the exposed group was less important (*n* = 378, 56%) than that in the unexposed group (*n* = 238, 68%). Less than one-third of the exposed mothers did household work with no direct income whereas nearly half of the unexposed mothers were involved in this type of occupation. The exposed group included more mothers doing income generating activity (*n* = 382, 57%) than the unexposed group (*n* = 140, 40%). Most mothers exposed to the program discussed child healthcare with their husband or child’s grandmother (*n* = 518, 77% against *n* = 171, 4 9% in the unexposed group). The majority of the exposed group reported reaching the nearest health center within 5 km from their residence. A toilet facility use was more frequent in the exposed group whereas the majority of the unexposed group had no toilet (*n* = 310, 88%). The proportion of those who have never done ANC in the unexposed group was three times larger than that of the exposed group.

**Table 1:** Characteristics of mothers of children under 24 months in Maradi and Zinder per program intervention exposure in Maradi and Zinder (*n* = 1026)

Variables	Exposed group		Unexposed group		
	*n*	*%*	*n*	*%*	*p* - value
*Socio-economic and demographic statu*					
Educational attainment					
no education	378	0.56	238	0.68	<.001
koranic school	145	0.22	76	0.22	
primary	79	0.12	20	0.06	
secondary or+	71	0.11	18	0.05	
Marital status					
monogamous	457	0.70	209	0.62	0.01
polygynous	196	0.30	127	0.38	
Parity					
primipara	74	0.11	43	0.12	0.56
multipara	599	0.89	309	0.88	
Occupation					
household work	211	0.31	162	0.46	<.001
agriculture/livestock	339	0.50	145	0.41	
sales/services	122	0.18	45	0.13	
Income generating activity	382	0.57	140	0.40	<.001
Who decide harvest use					
mother alone	235	0.41	85	0.35	0.12
husband	176	0.31	76	0.31	
mutual agreement	159	0.28	85	0.35	
Means of transport					
no	211	0.31	136	0.39	0.04
carriage	305	0.45	151	0.43	
vehicle	158	0.23	65	0.18	
Source of drinking water					
piped or public tap	154	0.23	63	0.18	0.06
borehole	290	0.43	169	0.48	
protected well	106	0.16	43	0.12	
traditional well	124	0.18	77	0.22	
Type of toilet facility					
no facility	487	0.72	310	0.88	<.001
traditional latrine	132	0.2	31	0.09	
ventilated/flush	54	0.08	10	0.03	
*Interactions with family and community members*					
Healthcare expenditure decision by mother alone	299	0.44	164	0.47	0.4
To whom asked for transport mean					
no one	129	0.33	75	0.36	0.8
parents	65	0.17	35	0.17	
NGO	65	0.17	37	0.17	
neighbors	130	0.33	62	0.30	
Discussed Key Family Practices^a^ with husband or child’s grand-mother	518	0.77	171	0.49	<.001
*Healthcare services*					
Accessed a health facility within 5 km	520	0.77	139	0.39	<.001
Number of antenatal visits					
Never	20	0.03	30	0.09	<.001
1–3 times	192	0.29	83	0.24	
> 4 times	455	0.68	236	0.68	
Type of personnel at ANC^b^					
doctor/nurse	268	0.41	146	0.46	0.18
midwife	382	0.59	173	0.54	

^a^Key Family Practices which address 8 thematic child health care that have been promoted by the behavior change communication program: exclusive breastfeeding until 6 month of age; sleeping under insecticide treated nets; washing hands with soap at critical moments; oral rehydration salt for diarrhea treatments; complementary feeding from 6 months of age; seeking preventive and curative care services; contraceptive methods for birth spacing

^b^Antenatal care

Overall bivariate analysis marked increase of early initiation of breastfeeding in the exposed group by 2.5 times (*n* = 394, 89%) compared to the unexposed group (*n* = 183, 77%). We examined the associations between the above cited characteristics of mothers and early the initiation of breastfeeding to identify which factors could influence behavior change in early breastfeeding. A few variables depicting the socio-economic and demographic status of mothers showed a significant association (Table 2): those who consulted their husband during harvest were 3.9 times more likely to initiate breastfeeding than those who made the decision alone (95% CI 1.8, 8.3). Those who had no access to a health facility within 5 km from their residence were 49% less likely to practice early breastfeeding (95% CI 0.3, 0.8). There was strong association between early breastfeeding and traditional latrine use (OR 7.9; 95% CI 2.5, 24.4) compared to no toilet use. Mothers who decided alone about healthcare expenditure were 2.2 times more likely to initiate breastfeeding in the first hour of birth compared to those letting others to make the decision (95% CI 1.4, 3.3). The odds of early breastfeeding among those who asked neighbors for transport to access the closest health center were greater than those without anyone to provide assistance (OR 4.3; 95% CI 1.9, 9.3). Discussing child healthcare with their husband was 2.4 times the odds of early breastfeeding compared that when discussion did not occur (95% CI 1.6, 3.7). Those who attended more than four antenatal care (ANC) visits were 2.8 times more likely to initiate breastfeeding within the first hour than those who have never attended ANC (95% CI 1.1, 7.1).

**Table 2:** Risk and protective factors associated with initiation of breastfeeding in Maradi and Zinder (crude odds) (*n* = 1026)

Variables	Initiation of breastfeeding within the first hour of birth		
	crude OR	95% CI	*p*-value
*Socio-economic and demographic status* Educational attainment			0.03
no education	1	–	
koranic school	0.50	0.31, 0.81	
primary	1.02	0.46, 2.25	
secondary or+	1.03	0.45, 2.40	
Marital status			0.46
monogamous	1	–	
polygynous	1.20	0.75, 1.89	
Parity			0.09
primipara	1	–	
multipara	1.62	0.92, 2.86	
Occupation			0.33
household work	1	–	
agriculture/livestock	1.68	0.84, 3.36	
sales/services	1.06	0.68, 1.66	
Income generating activity			0.88
no	1	–	
yes	1.03	0.68, 1.57	
Means of transport			0.03
no	1	–	
carriage	0.44	0.23, 0.84	
vehicle	0.45	0.23, 0.88	
Decision for harvest use by:			<.01
mother alone	1	–	
husband alone	1.28	0.75, 2.18	
both	3.90	1.84, 8.29	
Frequency of listening to radio			
not at all	1	–	0.65
once in the last 14 days	1.10	0.72, 1.70	
Source of drinking water			0.23
piped or public tap	1	–	
borehole	1.36	0.79, 2.34	
protected well	1.14	0.55, 2.37	
traditional well	0.78	0.43, 1.43	
Type of toilet facility			<.001
no facility	1	–	
traditional latrine	7.88	2.45, 25.36	
ventilated/flush	3.44	1.05, 11.30	
*Interactions with family and community members*			
Healthcare expenditure on child decision by:			<.001
other family members	1	–	
mother alone	2.16	1.41, 3.31	
To whom asked for assistance short of transport means			<.01
no one	1	–	
neighbors/friends	4.25	1.94, 9.30	
parents	2.07	0.92, 4.63	
rental/NGO	1.61	0.79, 3.29	
Discussed Key Family Practices with husband or child’s grandmother			<.001
no	1	–	
yes	2.39	1.56, 3.66	
*Healthcare services*			
Distance to health facility			<.01
within the village	1	–	
> 5 km	0.51	0.33, 0.77	
Number of antenatal visits			0.07
Never	1	–	
1–3 times	2.39	0.88, 6.46	
> 4 times	2.81	1.10, 7.14	
Type of personnel at ANC			
doctor/nurse	1	–	0.06
midwife	1.51	0.98, 2.32	

The program exposure was no longer significant when adjusted for socio-economic, demographic and behavioral factors (AOR 2.4; 95% CI 0.9, 6.2). We measured the adjusted odds of early breastfeeding in relation with key determinants identified in bivariate analysis (Table 3) such as educational attainment of mothers, means of transport to reach the nearest health facility and decision for harvest use. Type of occupation was also considered given that previous research proved its significant association with early breastfeeding. Mothers with a Koranic education were 60% less likely to initiate breastfeeding compared to those with no education (95% CI 0.2, 1.1). Those involved in sales or services were 7.7 times more likely to practice early breastfeeding compared to the household workers with no income (95% CI 1.3, 47.8). Mutual decision with their husband about harvest use showed 8.6 times the odds compared to sole decision by mothers (95% CI 2.0, 36.8).

**Table 3:** Risk and protective factors associated with initiation of breastfeeding. (adjusted odds) (*n* = 1026)

Variables	Initiation of breastfeeding within the first hour of birth		
	Adjusted OR^a^	95% CI	*p* - value
Educational attainment			
no education	1	–	–
koranic school	0.40	0.16, 1.05	0.06
school education^b^	0.66	0.23, 1.89	0.44
Occupation			
household work	1	–	–
agriculture/livestock	1.24	0.46, 3.34	0.67
sales/services	7.74	1.25, 47.84	0.03
Decision for harvest use by:			
mother alone	1	–	–
husband alone	2.25	0.74, 6.85	0.16
both	8.55	1.99, 36.8	<0.01
Use of toilet facility			
no	1	–	–
yes^c^	7.86	0.94, 65.9	0.06
Healthcare expenditure decision on child by:			
other family members	1	–	–
mothers alone	1.97	0.75, 5.18	0.17
To whom asked for assistance short of transport means			
no one	1	–	–
neighbors, family	4.86	0.84, 28.0	0.08
rent	0.86	0.19, 3.94	0.84
Discussed key family practices with their husband or child’s grand-mother:			
no	1	–	–
yes	0.94	0.37, 2.36	0.89
Distance to the health facility			
> 5 km	1	–	–
< 5 km	2.20	0.97, 4.99	0.06
Number of antenatal visits			
Never	1	–	–
1–3 times	1.72	0.25, 11.87	0.58
> 4 times	4.48	0.67, 29.85	0.12

^a^Odds ratio adjusted for the program exposure, region of residence, selected socio-demographic and economic variables: educational attainment of the interviewed mothers, type of occupation, decision for harvest use and means of transport

^b^School education is a merged category which combines educational attainment levels of primary, secondary and beyond

^c^Toilet facilities includes traditional and ventilated latrines

Among the selected independent variables thought to impact directly the timing of initiating breastfeeding after birth, independent decision of mothers about healthcare expenditure of their child, discussion with family members about child healthcare and number of antenatal care visits, revealed statistically no significant association with early breastfeeding. The odds of early breastfeeding was slightly significant among those who sought support from neighbors or their parents to obtain transport to access the nearest health facility compared to those who have no one to ask for such support (AOR 4.9; 95% CI 0.8, 28.0). Finally, with regard to healthcare services, those residing within 5 km from the nearest health facility showed weak evidence of its positive influence on early breastfeeding compared to those who accessed health services outside their village (AOR 2.2; 95% CI 1.0, 5.0). Toilet facility use can no longer be associated with early breastfeeding (AOR 7.9; 95% CI 0.9, 65.9).

## Discussion

This study has some limits that could bias the interpretation of the statistical findings. First, although we fully acknowledge the importance of considering the biologically beneficial effects of colostrum on both maternal and child health, when a child is given the first milk within the first hour of birth [22], the survey did not investigate mothers’ perception about colostrum. Second, given the design of the observational study which does not allow us to build a causal relationship between outcome and independent variables, the statistical findings of this study could not show evidence of what changed the mothers’ behavior with the early initiation of breastfeeding. We should therefore interpret these statistical findings with caution. We fully recognize the significance of a randomized control trial and strongly recommend it for the future impact assessment of the BCC program. Yet we deemed it important to use the available datasets of program evaluation surveys, which are often conducted in forms of observational studies and are not correctly interpreted despite considerable time and the funds invested.

Nevertheless, these findings could provide some clues to understand what could inhibit early initiation of breastfeeding. Highly significant association with social and economic vulnerability clearly indicates that early breastfeeding is impaired by poverty. They are characterized by poverty income, limited access to health facility and disintegration from family support and social network that compromises support from family, community and health professionals. Besides, autonomy of mothers able to take part in decision making about household budget distribution and to manage expenditure for their child’s health care positively influenced early initiation of breastfeeding. These findings allow the BCC program to revisit its strategy and focus their actions on the most deprived populations prevented from financial and technical support for improved environmental and maternal and child health.

The dataset includes unique sets of variables to address mothers’ behavior in family and community settings. At first sight, there is a significant difference in socio-economic and demographic characteristics of mothers between the two groups: exposed and unexposed to the program (Table 1). When comparing healthcare services related variables between the two groups, the chance of accessing health services within the village of residence is significantly greater in the intervention group. This is due to the non-randomization of the program intervention when selecting villages to start pilot BCC activities. The eligibility of villages was purposively defined based on the physical presence of a functional health center to be covered by the program. In short, socio-economically vulnerable mothers were excluded from the program interventions that targeted more educated groups bestowed with higher income opportunity and autonomy of decision making in household budget management.

A Koranic education revealed its negative influence on promoting early breastfeeding. This type of informal education is prone to marginalizing children and lacks openness to accept new behaviors. The author’s request to visit the masters of the Koranic Schools in Maradi region to discuss healthcare practice in a family was not approved. To our knowledge the BCC program did not associate Koranic school masters and it seems crucial to review profiles of communication channels in community settings and seek solutions when encountering opponents who are inclined to resist new behavior. The descriptive analysis by program exposure indicates that the BCC program was more focused on monogamous unions than polygynous unions. However we could not consider marital status as a confounder in this research: first there was no significant association between marital status and early breastfeeding in bivariate analysis. Second, further statistical analysis showed that polygynous mothers were better-off compared to monogamous. Previous studies associated polygyny with a high status of household economic wealth in Sub-Saharan Africa [23, 24].

Presence of husband and grand-mothers is often thought to be a risk factor for suboptimal breastfeeding, but the result of this study implies the opposite: when they are part of child health care through discussing and making decision together, there is positive impact on promoting breastfeeding. Yet this works only among the better off and better educated. Living with their husband and grandmothers does not necessarily mean that they are systematically supporting early breastfeeding and further qualitative research could bring insights to their expected roles to play within a family and community, and how they can be mobilized to become a promoter of early breastfeeding. Most village chiefs that we met on the ground expressed their satisfaction and pride of seeing mothers taking responsibility for their children in their village that became less ill and grew faster after the start of KFP promotion introduced by the BCC program. Besides, many community leaders reported having felt excluded from program activities, since these, according to them, involved exclusively women. The program could have further positive impact on early breastfeeding and possibly other KFP if mothers’ husband and child’s grandmother were involved in taking part and playing key roles in promoting optimal postpartum breastfeeding.

Although not significant statistically, we noticed positive influence of improved toilet use on early breastfeeding. This is illustrated by participatory Community-led Total Sanitation (CLTS) program implemented by UNICEF in parallel to child health care promotion. Villagers attended a group demonstration by a facilitator who mixed a glass of water with feces and asked them to drink it whereas they all refused systematically. They continued with a walk of shame to point out where they defecated the previous day. A causal chain between sanitation and frequent infectious diseases such as diarrhea became evident. Water feeding, which was a major cause of delayed initiation of breastfeeding during the first three days of birth, was no longer compatible with their children’s optimal health and growth. This behavior change occurred as the program successfully targeted suboptimum breastfeeding in a socio-cultural context where water for the majority of Muslim people is thought to have purifying effect. Besides hygiene and sanitation programs involved non-health factors within communities, such as school children and teachers, village leaders and those having critical decision-making roles in their society unlike the BCC program focused on health professionals, community workers and mothers. This example provides insights on how the BCC program promoting child healthcare should be designed to optimize its impact. Further research would be necessary to verify whether combined hygiene and breastfeeding promotion harnessed positive behavior change simultaneously.

Distance to reach the nearest health facility was an inhibitor to optimal breastfeeding and this implies that the BCC program did not extend equal positive effect on promoting early breastfeeding outside the health facility. This is partly because the BCC selected purposively villages with a health facility within accessible distance for its pilot activities. Another explanation would be that health professionals trained and supported by the program have a key role in leading mothers to initiating breastfeeding their child, and that grandmothers and traditional birth attendants who assist delivery at home were not brought in as communication channels for promoting early breastfeeding. Nevertheless, health professionals had limited impact on promoting early breastfeeding through antenatal care among the most vulnerable mothers who have no transport means to reach basic health services or reside outside health catchment area as ANC visits no longer influenced positively early initiation of breastfeeding.

Multivariate analysis revealed that the BCC program impact on early breastfeeding disappeared. The reasons why there is a discrepancy between the current study and the original article with regard to significance of the program impact on early breastfeeding could be explained by the following: The original article used a study sample in four regions (*n* = 2091) [17], whereas we conducted a secondary analysis of the same database using the sample in two regions (*n* = 1026) that were initially targeted by the program at the time of data collection. It is clear that the gap in the total number of study populations has led to a different result and, that with a smaller sample the program impact on early breastfeeding turned out to be no longer statistically significant.

## Conclusions

This study implies that the BCC program did not change early initiation of breastfeeding. Promoting early breastfeeding was impaired by social and economic vulnerability of mothers in rural Niger. Support from family and community members is a critical protective factor that contributed to mothers’ sustainable behavior change in early breastfeeding. The BCC program could further reach out to vulnerable mothers in early breastfeeding promotion by supporting an active involvement of family and community based non-health factors.

## Abbreviations

ANC: Antenatal care
BCC: Behavior change communication
KFP: Key family practices
ORS: Oral Rehydration Salt
